# Interaction between PVY HC-Pro and the NtCF_1_β-subunit reduces the amount of chloroplast ATP synthase in virus-infected tobacco

**DOI:** 10.1038/srep15605

**Published:** 2015-10-26

**Authors:** Yayi Tu, Yongsheng Jin, Dongyuan Ma, Heng Li, Zhenqian Zhang, Jiangli Dong, Tao Wang

**Affiliations:** 1 State Key Laboratory of Agrobiotechnology, College of Biological Sciences, China Agricultural University, Beijing 100193, China

## Abstract

The photosynthetic rate of virus-infected plants is always reduced. However, the molecular mechanism underlying this phenomenon remains unclear. The helper component-proteinase (HC-Pro) of *Potato virus Y* (PVY) was found in the chloroplasts of PVY-infected tobacco, indicating some new function of HC-Pro in the chloroplasts. We generated HC-Pro transgenic plants with a transit peptide to target the protein to chloroplast. The HC-Pro transgenic tobacco showed a decreased photosynthetic rate by 25% at the light intensity of 600 μmol m^−2^ s^−1^. Using a yeast two-hybrid screening assay to search for chloroplast proteins interacting with HC-Pro, we identified that PVY HC-Pro can interact with the chloroplast ATP synthase NtCF_1_β-subunit. This interaction was confirmed by GST pull-down and co-immunoprecipitation assays. HC-Pro didn’t interfere with the activity of assembled ATP synthase *in vitro*. The HC-Pro/NtCF_1_β-subunit interaction might affect the assembly of ATP synthase complex. Quantitative western blot and immunogold labeling of the ATP synthase indicated that the amount of ATP synthase complex was decreased in both the HC-Pro transgenic and the PVY-infected tobacco. These results demonstrate that HC-Pro plays an important role in reducing the photosynthetic rate of PVY-infected plants, which is a completely new role of HC-Pro besides its multiple known functions.

*Potato virus Y* (PVY) is a single-stranded, positive-sense RNA virus of the genus *Potyvirus* and enters the top 10 plant virus list for the journal *Molecular Plant Pathology*^1^. Viral infection causes symptoms, such as chlorosis and necrosis, that are associated with changes in chloroplast structure and function^2^,^3^,^4^,^5^,^6^,^7^. The net photosynthetic rate is significantly reduced in virus-infected plants^6^,^8^,^9^,^10^.

Photosynthesis is the basis for plant growth and represents a primary target of viral infection^11^. Photosynthetic electron transport is coupled to ATP synthesis through the process of photophosphorylation^12^. The proton motive force (pmf) across the thylakoid membrane is used by the chloroplast ATP synthase to generate ATP for CO_2_ fixation. Chloroplast ATP synthase exists as a multi-subunit complex with distinct stromal and transmembrane regions known as CF_1_ and CF_o_, respectively^13^,^14^,^15^. The CF_o_ part is an integral membrane-spanning proton transport protein complex, and the CF_1_ part contains the catalytic sites for reversible ATP synthesis^16^. The CF_1_β-subunit contains a single nucleotide-binding site, while in the α_3_β_3_ hexamer, the nucleotide-binding sites are located at each of the six α-β-subunit interfaces^17^. The catalytic sites are thought to be located primarily on the β-subunits, while the regulatory sites are found on the α-subunits^18^. The three β-subunits are believed to proceed sequentially through conformational changes that facilitate the ATP binding, interconversion and release steps^19^. The N-terminus of the β-subunit is important for its assembly into the CF_1_ complex^20^.

The helper component proteinase (HC-Pro) is one of the 11 mature proteins encoded by potyvirus^21^,^22^. HC-Pro is a multifunctional protein with several suggested roles in the viral infection cycle^23^, including its involvement in aphid transmission^24^,^25^, viral cell-to-cell and long-distance movement^26^,^27^,^28^, polyprotein processing^29^ and suppression of post-transcriptional gene silencing (PTGS) in plants^30^,^31^,^32^. Many interactions between host factors and viral gene products have been found using a yeast two-hybrid system^33^. Various host factors have been found to interact with HC-Pro, including a calmodulin-related protein^34^; two novel RING finger proteins, HIP1 and HIP2^35^; three 20S proteasome subunits, PAA, PBB and PBE^36^; and a calreticulin found in papaya^37^. The tobacco chloroplast protein NtMinD was first reported to interact with PVY HC-Pro in 2007^38^, and the HC-Pro of *Sugar cane mosaic virus* (ScMV) has been found to interact with maize ferredoxin-5^39^. HC-Pro is also involved in the modulation of host proteosomal catalytic activity^40^. In 2011, HC-Pro from the genus *Potyvirus* was shown to interact with the translation initiation factor eIF4E and its isoform eIF(iso)4E^41^. PVA HC-Pro can also interact with the microtubule-associated host protein HIP2^42^, and mutations in a highly variable region of PVA HC-Pro can affect this interaction^43^. Furthermore, HC-Pro interacts with an ethylene-inducible transcription factor to block RNA silencing^44^. Meanwhile, a calmodulin-like protein in tobacco can bind to HC-Pro and direct the degradation of the viral RNA silencing suppressors (RSS) to enhance the host antiviral RNAi^45^.

PVY HC-Pro was reported to be present in the leaf chloroplasts of virus-infected plants^46^, and this result was confirmed in our present work. The mechanism that HC-Pro uses to enter the chloroplasts remains unclear, but we are interested in the function of HC-Pro in the chloroplasts. Here, we show that PVY HC-Pro interacted with the chloroplast NtCF_1_β-subunit both *in vitro* and *in vivo*. The HC-Pro/NtCF_1_β-subunit interaction led to a decreased amount of chloroplast ATP synthase in both the HC-Pro transgenic and the PVY-infected tobacco, suggesting a new role of PVY HC-Pro in the disturbance of the photosynthesis of virus-infected plants.

## Results

### The photosynthetic rate of PVY-infected plants was reduced

**Figure 1:**
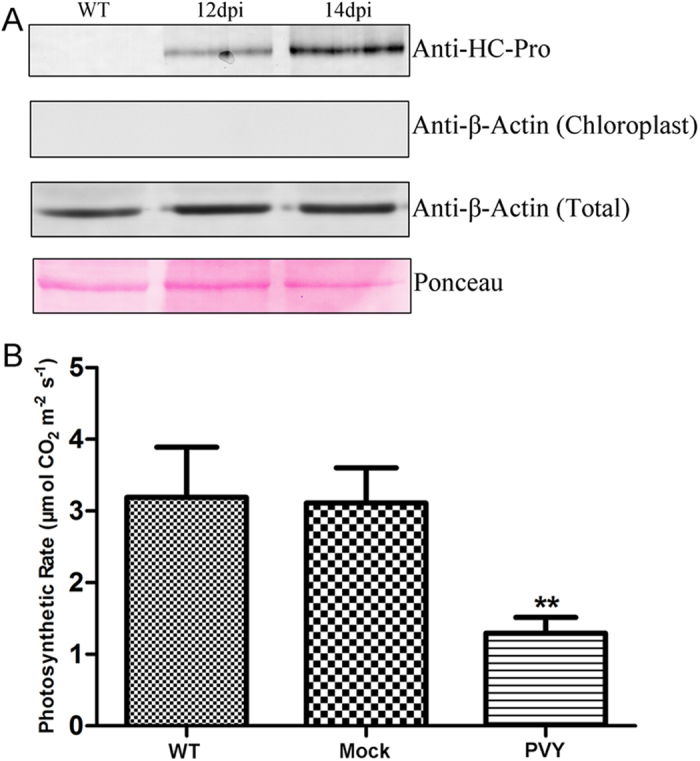
The photosynthetic rate was reduced in PVY-infected plants. (**A**) Detection of HC-Pro in the chloroplasts of wild-type (WT) and PVY-infected tobacco at 12 and 14 days post inoculation (dpi). Antibody raised against HC-Pro (Anti-HC-Pro) was used to detect HC-Pro accumulation in the chloroplasts (top panel). The β-Actin was not detected in the chloroplast proteins (second panel). The detection of β-Actin in total leaf proteins verified the effectivity of the Anti-β-Actin antibody (third panel). Ponceau staining of the large subunit of Rubisco was used as a loading control (bottom panel). (**B**) Photosynthetic rate of wild-type (WT), mock-inoculated (Mock) and PVY-infected (PVY) tobacco at the light intensity of 600 μmol m^−2^ s^−1^. The photosynthetic rate of PVY-infected plants was significantly reduced by 58% compared with that of the wild-type plants (Student’s t-test, **P < 0.01). There was no difference between the wild-type and mock-inoculated plants (Student’s t-test, P > 0.05). Three biological repeats were done.

PVY HC-Pro was reported to be present in the chloroplasts of virus-infected plants^46^ and we confirmed this result in our present work. Wild-type tobacco was inoculated with PVY viruses and intact chloroplasts of PVY-infected plants were isolated. HC-Pro was detected at 12 to 14 days post inoculation (dpi) in the chloroplasts of PVY-infected plants with an anti-HC-Pro antibody raised against the peptide DYRRQPGVSRKCTSSKDGN in rabbit. The purity of isolated chloroplast was examined by western-blot detection of the widely expressed cytoplasmic protein β-Actin. The β-Actin was detected in the total leaf proteins, but not in the purified chloroplasts (Fig. 1A). The results demonstrated that there was no contamination of cytoplasmic proteins in our purified chloroplasts. Meanwhile, we determined the photosynthetic rate in wild-type, mock-inoculated and PVY-infected plants at 12 dpi at the light intensity of 600 μmol m^−2^ s^−1^. The results showed that the photosynthetic rate of PVY-infected plants was reduced by 58% compared with that of wild-type plants, and the mock-inoculated plants presented a similar photosynthetic rate to the wild-type plants (Fig. 1B).

### The photosynthetic rate of HC-Pro transgenic plants was reduced

**Figure 2:**
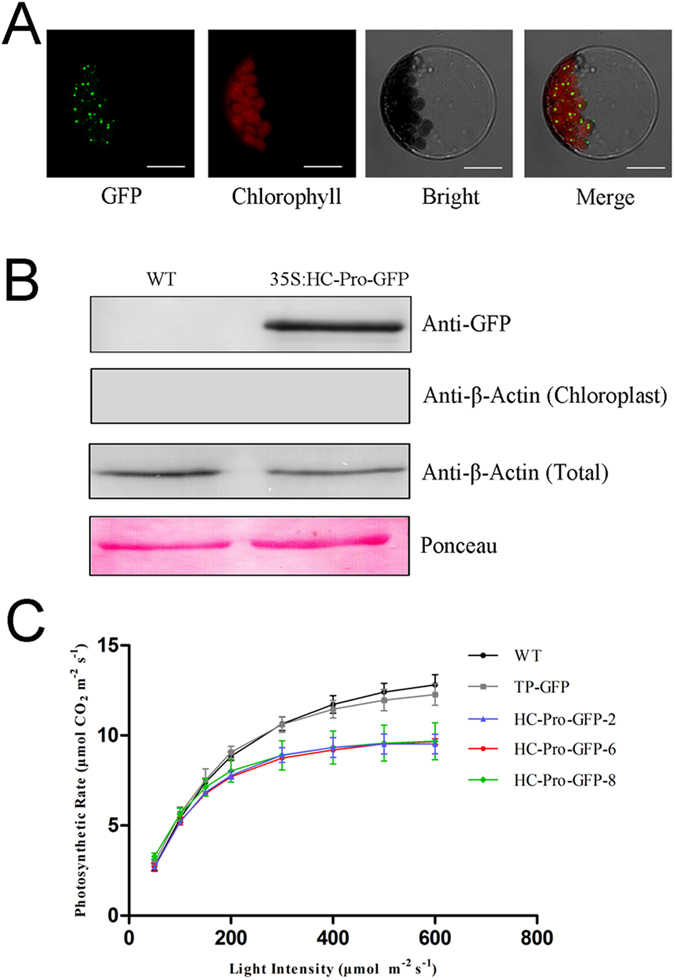
The photosynthetic rate of HC-Pro transgenic plants was reduced. (**A**) Subcellular localization of HC-Pro in *Arabidopsis* protoplast with the transit peptide. The transit peptide of the ribulose bisphosphate carboxylase small subunit (*rbcs*) was fused to the N-terminus of HC-Pro. Arabidopsis protoplasts were transformed with a construct carrying TP-HC-Pro-GFP under the control of the cauliflower mosaic virus (CaMV) 35S promoter. The GFP field indicated the subcellular localization of HC-Pro in the chloroplasts. All scale bars indicate 5 μm. (**B**) Western-blot of chloroplast protein extracts from wild-type and HC-Pro transgenic plants. The chloroplast proteins were extracted from intact chloroplasts of wild-type (WT) and HC-Pro transgenic plants (35S:HC-Pro-GFP). Anti-GFP was used to detect the HC-Pro-GFP fusion protein (top panel). The β-Actin was not detected in the chloroplast proteins (second panel). The detection of β-Actin in total leaf proteins verified the effectivity of the Anti-β-Actin antibody (third panel). Ponceau staining of the large subunit of Rubisco was used as a loading control (bottom panel). (**C**) Photosynthesis in the wild-type (WT), empty-vector transgenic (TP-GFP) and the HC-Pro transgenic (HC-Pro-GFP) plants. The photosynthetic rate of the HC-Pro transgenic plants was decreased compared to the wild-type plants, while plants transformed with the empty vector demonstrated a photosynthetic rate similar to that of the wild-type plants. Three lines were used and there biological repeats were done for each line.

We hypothesized that HC-Pro might interact with the host components involved in photosynthesis. To test this hypothesis, we generated transgenic plants retargeting HC-Pro to the chloroplasts to focus on the function of HC-Pro in chloroplasts. The transit peptide of the ribulose bisphosphate carboxylase small subunit (*rbcs*) was fused to the N-terminus of HC-Pro to target the protein to chloroplasts. The subcellular location of HC-Pro in the protoplasts of *Arabidopsis* demonstrated that the transit peptide functioned efficiently to lead HC-Pro into the chloroplasts (Fig. 2A). Thus, the transit peptide was used to generate transgenic plants with HC-Pro retargeted to chloroplasts. Meanwhile, HC-Pro was fused to a GFP tag at its C-terminus to form a HC-Pro-GFP fusion. Plants transformed with the HC-Pro-GFP fusion retargeted to chloroplasts were referred to as HC-Pro transgenic plants. Intact chloroplasts of HC-Pro transgenic plants were isolated and the accumulation of HC-Pro in the chloroplasts of transgenic tobacco was detected with an anti-GFP antibody. The purity of isolated chloroplast was examined by western-blot detection of the widely expressed cytoplasm protein β-Actin (Fig. 2B). We also generated transgenic plants with the empty vector that contained only the transit peptide and the GFP tag, which was referred to as empty-vector transgenic plants, as a control for HC-Pro transgenic plants in addition to the wild-type control. The photosynthetic rates of the wild-type, empty-vector transgenic and the HC-Pro transgenic plants were then determined. The photosynthetic rate of the HC-Pro transgenic plants was reduced by 25% on average compared to that of the wild-type plants at the light intensity of 600 μmol m^−2^ s^−1^, while plants transformed with the empty vector presented a similar photosynthetic rate as the wild-type plants (Fig. 2C).

### PVY HC-Pro and the NtCF_1_β-subunit interacted in yeast cells

The reduction in the photosynthetic rate of the HC-Pro transgenic plants was in accordance with the reduced photosynthesis in PVY-infected plants, indicating that HC-Pro participated in the disturbance of photosynthesis in virus-infected plants. Therefore, we used a yeast two-hybrid screen to identify proteins that may interact with PVY HC-Pro. We identified HC-Pro-interacting proteins from a *Nicotiana tabacum* cDNA library constructed by our lab using the tobacco leaves^38^. Among the obtained positive clones, one encoded a polypeptide containing 208 amino acids that shared 100% identity with the tobacco chloroplast CF_1_β-subunit. The full-length gene sequence of the tobacco CF_1_β-subunit (*NtCF*_*1*_*β-subunit*) was cloned into the pMD18-T vector (Takara) and verified by sequencing. Based on sequence alignment, the cloned sequence exhibited 100% identity with a tobacco chloroplast DNA sequence (GenBank accession no: Z00044.2) on file in GenBank.

**Figure 3:**
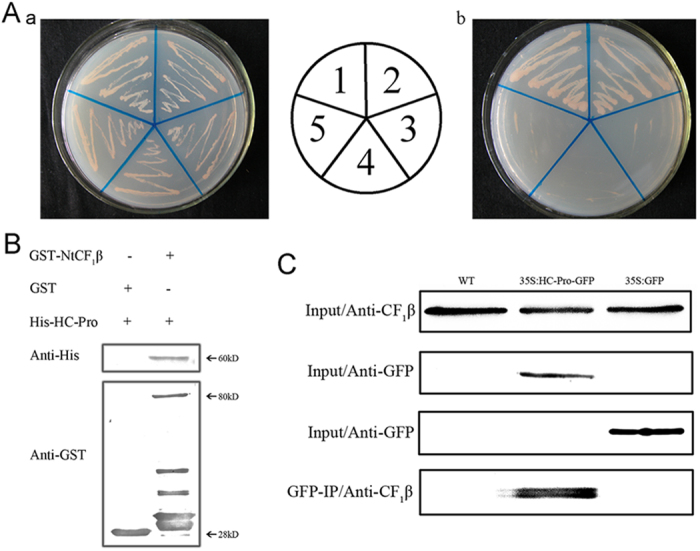
HC-Pro interacted with the NtCF_1_β-subunit. (**A**) Yeast two-hybrid assay of HC-Pro and the NtCF_1_β-subunit in transformed *S. cerevisiae* AH109 cells grown on SD/-Leu/-Trp (a) and on SD/-Ade/-His/-Leu/-Trp (b). 1, pGBKT7–53/pGADT7-RecT (positive control); 2, pGBKT7-HC-Pro/pGADT7-NtCF_1_β-subunit; 3, pGBKT7/pGADT7 (negative control); 4, pGBKT7-HC-Pro/pGADT7; 5, pGBKT7/pGADT7-NtCF_1_β-subunit. (**B**) GST-pull down assay of HC-Pro and the NtCF_1_β-subunit. His-HC-Pro was pulled down by the GST-NtCF_1_β protein (right lane) but not by the GST protein alone (left lane). The GST-NtCF_1_β fusion protein was 80 kDa. The His-HC-Pro fusion protein was 60 kDa. The GST tag was 28 kDa. The molecular weights of these proteins are indicated on the right side of the blot. (**C**) Co-immunoprecipitation assay of HC-Pro and the NtCF_1_β-subunit in isolated intact chloroplasts *in vivo*. The NtCF_1_β-subunit could be co-immunoprecipitated with HC-Pro in chloroplasts from HC-Pro transgenic plants (35S:HC-Pro-GFP) but not in the wild-type plants (WT) or the empty-vector transgenic plants (35S:GFP). The inputs of NtCF_1_β subunit, HC-Pro-GFP and GFP tag were shown on the three upper panels. The GFP-IP result was shown on the bottom panel.

We previously inserted the full-length coding sequence of HC-Pro in-frame into the GAL4 DNA-binding domain of pGBKT7^36^. Here, the full-length coding sequence of the NtCF_1_β-subunit was inserted into the GAL4 activation domain of pGADT7. The reconstructed plasmid, pGADT7-NtCF_1_β, was co-transformed with pGBKT7-HC-Pro into *Saccharomyces cerevisiae* AH109 cells. The pGBKT7-HC-Pro/pGADT7-NtCF_1_β transformant was able to grow on synthetic defined (SD) medium lacking Ade, His, Leu and Trp (SD/-Ade/-His/-Leu/-Trp), as did the positive control pGBKT7–53/pGADT7-RecT, suggesting that PVY HC-Pro could interact with NtCF_1_β in yeast cells (Fig. 3A).

### The interaction between HC-Pro and the NtCF_1_β-subunit was further verified both *in vitro* and *in vivo*

To verify the interaction between HC-Pro and the NtCF_1_β-subunit, a pull-down assay was employed. The HC-Pro and NtCF_1_β-subunit proteins were expressed in *E. coli* and then we purified them. The NtCF_1_β-subunit was fused to a GST tag, and HC-Pro was expressed with a 6 × His tag. The GST-tagged NtCF_1_β-subunit was bound to Glutathione Sepharose 4B and incubated with His-HC-Pro. After washing the beads, His-HC-Pro was detected with an anti-His antibody. HC-Pro was pulled down by NtCF_1_β-subunit but not the GST tag alone (Fig. 3B). The pull-down assay confirmed the interaction between HC-Pro and the NtCF_1_β-subunit *in vitro*.

To confirm the interaction between HC-Pro and NtCF_1_β-subunit *in vivo*, intact chloroplasts from wild-type, empty-vector transgenic and HC-Pro transgenic plants were isolated, and a co-immunoprecipitation assay was performed using the chloroplast proteins. Because PVY HC-Pro was fused to a GFP tag at its C-terminus in the transgenic plants, an anti-GFP antibody was used to precipitate the HC-Pro/NtCF_1_β-subunit complex. The results showed that the NtCF_1_β-subunit co-immunoprecipitated with HC-Pro in the chloroplasts of transgenic plants *in vivo* but not in the chloroplasts of both the wild-type and empty-vector transgenic plants (Fig. 3C), thus demonstrating the interaction between HC-Pro and the NtCF_1_β-subunit in chloroplasts of living cells.

### Residues 1–97 of PVY HC-Pro and residues 1–95 of NtCF_1_β-subunit were necessary for HC-Pro/NtCF_1_β-subunit interaction

**Figure 4:**
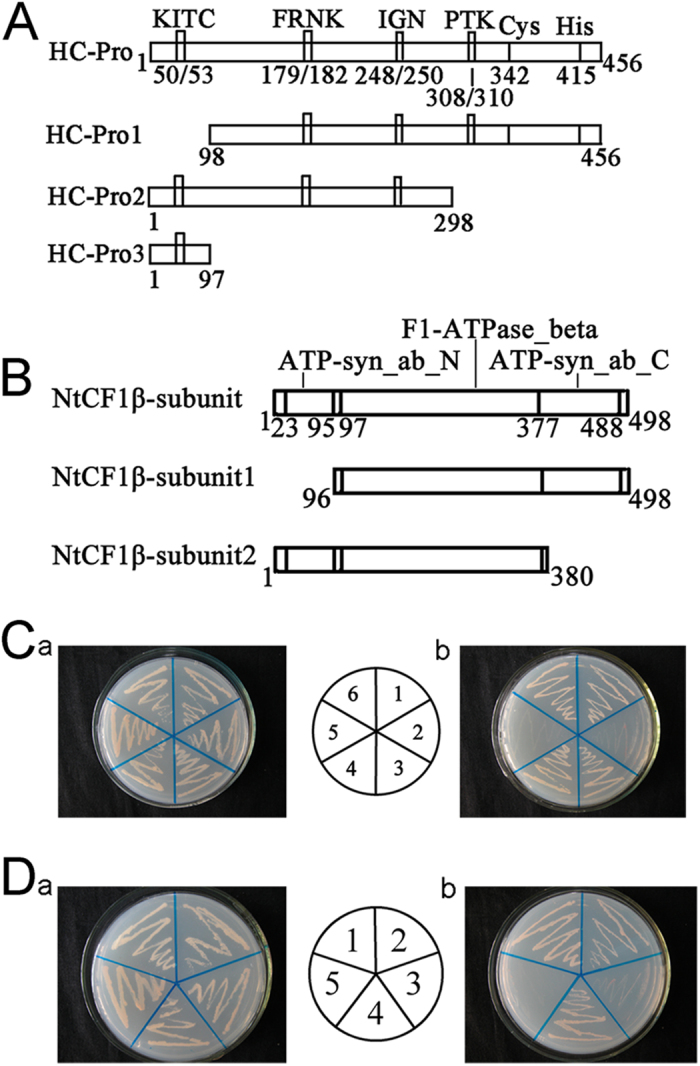
Necessary domains for HC-Pro/NtCF_1_β-subunit interaction. (**A**) Schematic overview of the HC-Pro domains and deletion mutants. The N-terminal mutant lacks amino acids 1–97, and the C-terminal mutant lacks amino acids 299–456. HC-Pro3 is a mutant containing only the 97 N-terminal amino acids. The mutants were designed to identify the domains of HC-Pro that interact with the NtCF_1_β-subunit. (**B**) Schematic overview of the NtCF_1_β-subunit domains and deletion mutants. The N-terminal mutant lacks amino acids 1–95, and the C-terminal mutant lacks amino acids 381–498. The mutants were designed to identify the domains of NtCF_1_β-subunit that interact with HC-Pro. (**C**) Interactions between the PVY HC-Pro mutants and the full-length NtCF_1_β-subunit in transformed *S. cerevisiae* AH109 cells grown on SD/-Leu/-Trp (a) and on SD/-Ade/-His/-Leu/-Trp (b). 1, pGBKT7-HC-Pro/pGADT7-NtCF_1_β-subunit; 2, pGBKT7-HC-Pro1/pGADT7-NtCF_1_β-subunit; 3, pGBKT7-HC-Pro2/pGADT7-NtCF_1_β-subunit; 4, pGBKT7-HC-Pro3/pGADT7-NtCF_1_β-subunit; 5, pGBKT7/pGADT7 (negative control); 6, pGBKT7–53/pGADT7-RecT (positive control). The results indicated that the N-terminus of HC-Pro (amino acids 1–97) was necessary for its interaction with the NtCF_1_β-subunit. (**D**) Interactions of full-length PVY HC-Pro and the mutants of NtCF_1_β-subunit in transformed *S. cerevisiae* AH109 cells grown on SD/-Leu/-Trp (a) and on SD/-Ade/-His/-Leu/-Trp (b). 1, pGBKT7–53/pGADT7-RecT (positive control); 2, pGBKT7-HC-Pro/pGADT7-NtCF_1_β-subunit; 3, pGBKT7-HC-Pro/pGADT7-NtCF_1_β-subunit1; 4, pGBKT7-HC-Pro/pGADT7-NtCF_1_β-subunit2; 5, pGBKT7/pGADT7 (negative control). The results indicated that the N-terminus of the NtCF_1_β-subunit (amino acids 1–95) was necessary for its interaction with HC-Pro.

The above results confirmed that the NtCF_1_β-subunit could interact with PVY HC-Pro both *in vivo* and *in vitro.* We further explored the interaction domains of the two proteins. To investigate the domains necessary for PVY HC-Pro to interact with the NtCF_1_β-subunit, we employed the following three previously designed deletion mutants for PVY HC-Pro: HC-Pro1 (residues 98–456), HC-Pro2 (residues 1–298) and HC-Pro3 (residues 1–97)^36^ (Fig. 4A). And two deletion mutants for the NtCF_1_β-subunit, NtCF_1_β-subunit1 (residues 96–498) and NtCF_1_β-subunit2 (residues 1–380), were designed to investigate the domains necessary for the NtCF_1_β-subunit to interact with PVY HC-Pro (Fig. 4B).

The full-length NtCF_1_β-subunit and the HC-Pro mutants were co-transformed into yeast cells. The HC-Pro1/NtCF_1_β-subunit transformant was not able to grow on SD/-Ade/-His/-Leu/-Trp, but the HC-Pro2/NtCF_1_β-subunit and HC-Pro3/NtCF_1_β-subunit transformants were able to grow on this medium (Fig. 4C). These results indicated that the N-terminal region of PVY HC-Pro (residues 1–97) was necessary for its interaction with the NtCF_1_β-subunit.

The full-length HC-Pro and the NtCF_1_β-subunit mutants were co-transformed into yeast cells. In the yeast two-hybrid system, the NtCF_1_β-subunit2/HC-Pro transformant was able to grow on SD/-Ade/-His/-Leu/-Trp, whereas the NtCF_1_β-subunit1/HC-Pro transformant was not (Fig. 4D). Hence, residues 1–95 of the NtCF_1_β-subunit were necessary for the interaction with PVY HC-Pro.

### PVY HC-Pro had no effect on the enzymatic activity of ATP synthase

**Figure 5:**
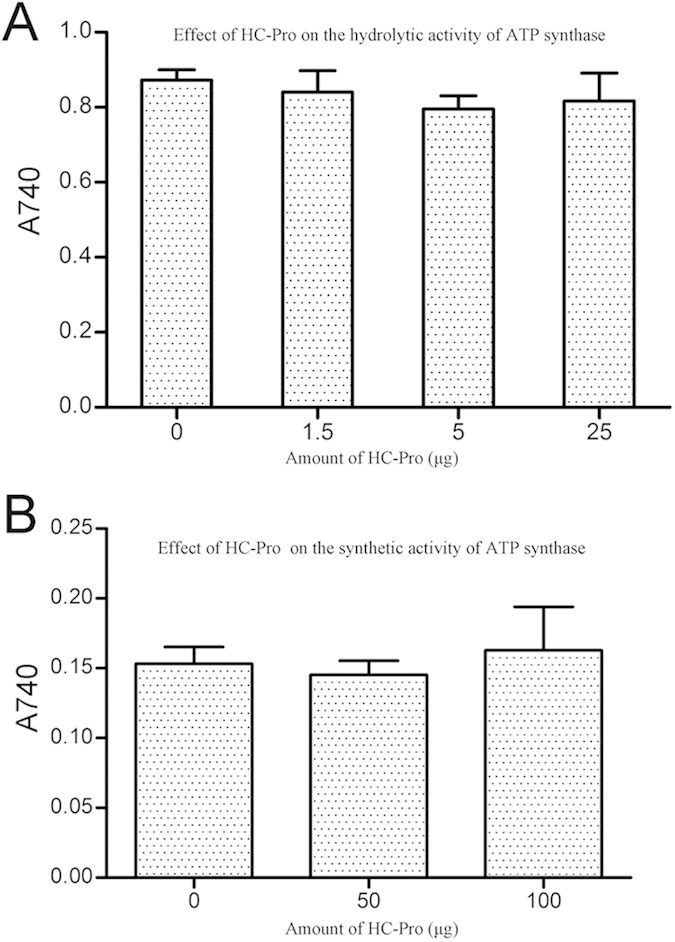
HC-Pro has no effect on the enzymatic activity of ATP synthase. (**A**) Measurement of the hydrolytic activity of ATP synthase influenced by HC-Pro. Different amounts of purified HC-Pro (indicated on the X-axis as 0 μg, 1.5 μg, 5 μg and 25 μg) were added to the same amount of isolated CF_1_ complex (5 ug). The A_740_ values on the Y-axis represented the amount of Pi released from the hydrolytic reaction catalyzed by CF_1_. The hydrolytic activity of isolated CF_1_ was not affected by the addition of purified HC-Pro (Student’s t-test, P > 0.05). (**B**) Measurement of the synthetic activity of ATP synthase influenced by HC-Pro. Different amounts of purified HC-Pro (indicated at the X-axis as 0 μg, 50 μg and 100 μg) were added into the same amount of active thylakoid membrane (20 ug). The A_740_ values indicated along the Y-axis represented the amount of Pi remaining in the reaction mix after the ATP synthetic reaction. The synthetic activity of ATP synthase was not disturbed by the interaction between HC-Pro and the NtCF_1_β-subunit (Student’s t-test, P > 0.05).

To further elucidate the biological significance of the interaction between HC-Pro and the NtCF_1_β-subunit, we purified PVY HC-Pro and investigated its effect on the enzymatic activity of ATP synthase. The CF_1_ complex of ATP synthase from spinach was isolated and its ability to hydrolyze ATP was examined. Purified HC-Pro was added to the CF_1_ complex to test its influence on the enzyme activity of ATP synthase. The hydrolytic activity of ATP synthase was not disturbed by purified HC-Pro (Fig. 5A). There were no significant differences between the activity of CF_1_ alone and CF_1_ with the HC-Pro protein (Student’s t-test, P > 0.05).

Active thylakoid membranes were extracted from spinach and the ATP synthetic activity was examined. Purified HC-Pro was added to the membrane extract to examine its influence on the synthetic activity of ATP synthase. Again, no disturbance in the synthetic activity was observed (Fig. 5B). There were no significant differences between the synthetic activity of ATP synthase alone and ATP synthase with the HC-Pro protein (Student’s t-test, P > 0.05).

### The content of ATP synthase was reduced in HC-Pro transgnic plants

The *in vitro* biochemical reaction suggested that HC-Pro did not interfere with the enzymatic activity of chloroplast ATP synthase. Because CF_1_ is a complex with several subunits, we assumed that the interaction between HC-Pro and the NtCF_1_β-subunit might affect the assembly of the CF_1_ complex and could thus reduce the amount of ATP synthase in HC-Pro transgenic plants. To test this hypothesis, both quantitative western blot and immunogold labeling methods were used to determine the amount of ATP synthase.

**Figure 6:**
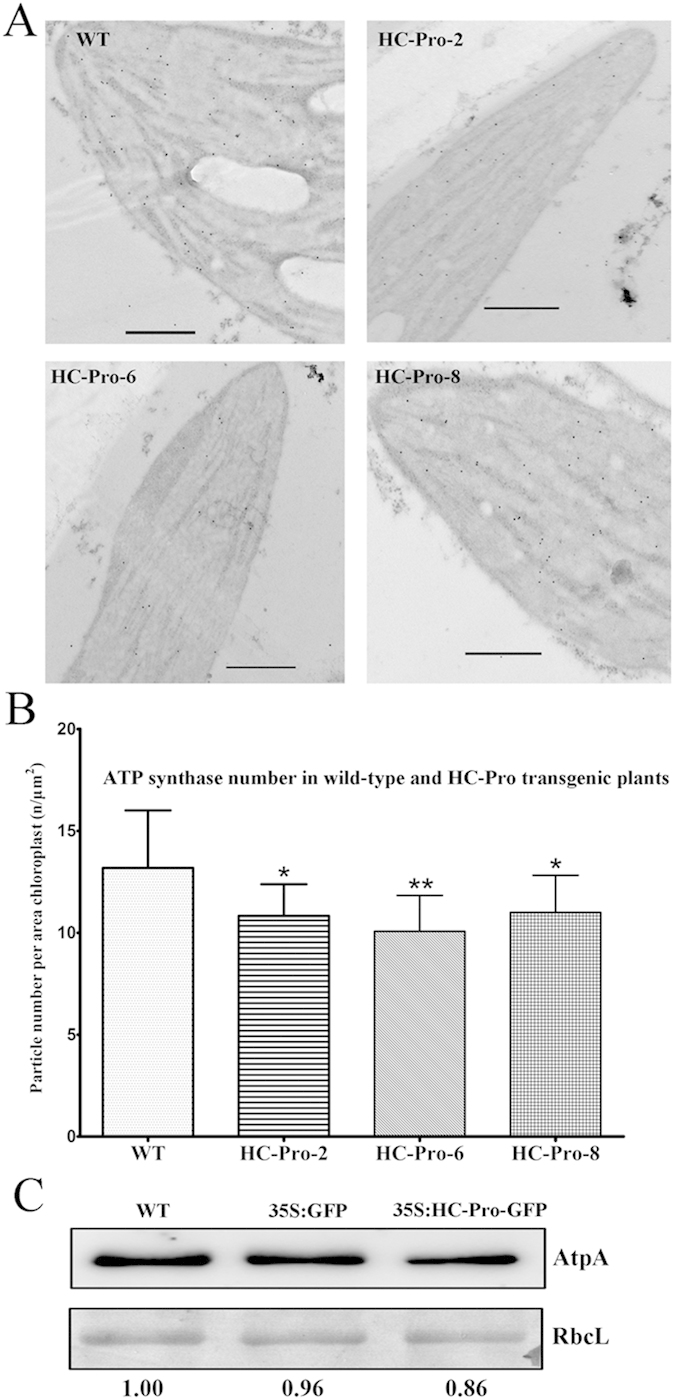
The level of ATP synthase was reduced in HC-Pro transgenic plants. (**A**) Typical immunogold labeling TEM images of wild-type (WT) and HC-Pro transgenic tobacco (HC-Pro-2, HC-Pro-6, HC-Pro-8). The 10-nm gold particles represent the chloroplast ATP synthase. Scale bars indicate 500 nm. (**B**) Statistical data of the number of ATP synthase molecules in wild-type and HC-Pro transgenic plants. The number of ATP synthase complexes per area of the chloroplast was used to compare the different levels of ATP synthase between wild-type (WT) and HC-Pro transgenic (HC-Pro-2, HC-Pro-6, HC-Pro-8) plants. The amount of ATP synthase was reduced by 16% on average in the HC-Pro transgenic plants (Student’s t-test, *P < 0.05, **P < 0.01). Three lines were examined and ten sections were used for three biological replicates for each line. More than 20 chloroplasts were counted with each sample. (**C**) Quantitative western-blot of ATP synthase. Intact chloroplasts were isolated and chloroplast proteins were loaded for western-blot analysis. ATP synthase abundance was determined from the contents of the essential α-subunit of CF_1_. Anti-AtpA was used to detect the CF_1_ α-subunit. Estimates of AtpA protein levels relative to the large subunit of Rubisco (RbcL) in the empty-vector transgenic plants (35S:GFP) and the HC-Pro transgenic plants (35S:HC-Pro-GFP) compared with those in wild-type plants (WT) were shown at the bottom.

First, we calculated the number of ATP synthase molecules in wild-type and HC-Pro transgenic plants. Typical immunogold labeling TEM images of wild-type plants and HC-Pro transgenic plants were shown (Fig. 6A). The amount of ATP synthase was found to be reduced by 16% on average in HC-Pro transgenic tobacco compared with the wild-type plants (Student’s t-test, *P < 0.05, **P < 0.01) (Fig. 6B). Meanwhile, intact chloroplasts of the wild-type, empty-vector transgenic and HC-Pro transgenic plants were isolated and chloroplast proteins were extracted. The content of ATP synthase was determined by quantitative western blot using an antibody against the essential α-subunit of CF_1_ (AtpA). The large subunit of Rubisco (RbcL) was used as a loading control to demonstrate equal input in all lanes. The amount of ATP synthase in HC-Pro transgenic plants was reduced by 14% compared to the wild-type plants and the amount of ATP synthase in empty-vector transgenic plants was nearly the same as the wild-type plants (Fig. 6C). The levels of the AtpA and RbcL proteins were quantified using ImageJ software.

### The content of ATP synthase was reduced in PVY-infected plants

**Figure 7:**
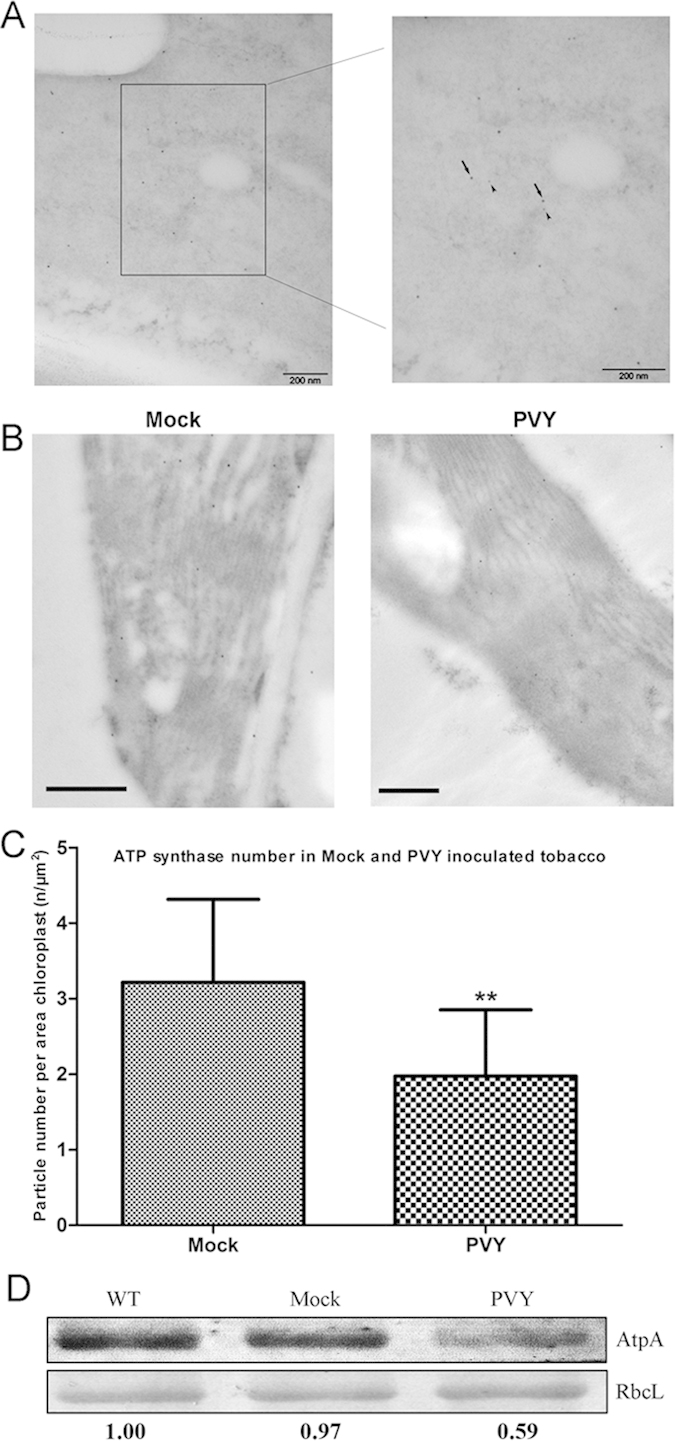
The number of ATP synthase molecules was reduced in PVY-infected plants. (**A**) Co-localization of HC-Pro and NtCF_1_β-subunit in PVY-infected plants. HC-Pro was labeled by 10-nm gold particles and NtCF_1_β-subunit was labeled by 5-nm gold particles. Arrows indicated the localization of HC-Pro in the chloroplasts. Arrow heads indicated the localization of NtCF_1_β-subunit. The adjacent position of the 10-nm and 5-nm gold particles indicated the co-localization of HC-Pro and NtCF_1_β-subunit. Scale bars represent 200 nm. (**B**) Typical immunogold labeling TEM images of mock-inoculated (Mock) and PVY-infected tobacco (PVY). The 10-nm gold particles represent the chloroplast ATP synthase. Scale bars indicate 400 nm. (**C**) Statistical data of the number of ATP synthase molecules in mock-inoculated (Mock) and PVY-infected (PVY) tobacco. The number of ATP synthase molecules per area of the chloroplast was employed for comparison of the differences between mock-inoculated and PVY-infected plants. Ten sections were used for three biological replicates. Calculations were performed for over 15 chloroplasts with each sample. The quantity of ATP synthase was found to be significantly reduced, by 39% on average, in the PVY-infected plants (Student’s t-test, **P < 0.01). (**D**) Quantitative western-blot of ATP synthase. Intact chloroplasts were isolated and chloroplast proteins were loaded for western-blot analysis. ATP synthase abundance was determined from the contents of the essential α-subunit of CF_1_. Anti-AtpA was used to detect the CF_1_ α-subunit. Estimates of AtpA protein levels relative to the large subunit of Rubisco (RbcL) in the mock-inoculated plants (Mock) and the PVY-infected plants (PVY) compared with those in wild-type plants (WT) were shown at the bottom.

The above results showed that the interaction between HC-Pro and the NtCF_1_β-subunit led to a reduced amount of ATP synthase in HC-Pro transgenic plants. To verify this important interaction in natural viral infection, we used electron microscopy analysis to determine the co-localization of HC-Pro and the NtCF_1_β-subunit in PVY-infected tobacco. PVY HC-Pro was labeled with 10-nm gold particles and the NtCF_1_β-subunit was labeled with 5-nm gold particles. Electron microscopy analysis showed that PVY HC-Pro and NtCF_1_β-subunit co-localized in the chloroplasts of PVY-infected tobacco (Fig. 7A). And we also calculated the number of ATP synthase molecules in mock and PVY-inoculated tobacco. Typical immunogold labeling TEM images were presented (Fig. 7B). The amount of ATP synthase was observed to be significantly reduced, by 39% on average, in PVY-infected tobacco compared with the mock-inoculated plants (Student’s t-test, **P < 0.01) (Fig. 7C). Intact chloroplasts of the wild-type, mock-inoculate and PVY-infected plants were isolated and the content of ATP synthase was also determined using an antibody against the essential α-subunit of CF_1_ (AtpA). The large subunit of Rubisco (RbcL) was used as a loading control to demonstrate equal input in all lanes. The amount of ATP synthase in PVY-infected plants was diminished by 41% compared to the wild-type plants and the amount of ATP synthase in mock-inoculated plants was nearly the same as the wild-type plants (Fig. 7D). The levels of the AtpA and RbcL proteins were quantified using ImageJ software.

The above results showed that the interaction of HC-Pro and the NtCF_1_β-subunit did not interfere with the enzymatic activity of ATP synthase but instead decreased the amount of ATP synthase in both HC-Pro transgenic plants and PVY-infected plants. Our results showed a new function of PVY HC-Pro in viral attacks on the photosynthetic system of plants.

## Discussion

Viral infection is a complicated process that involves both viral components and host factors. As demonstrated in the integrated molecular model of plant-virus interactions presented by Elena *et al.*^47^, the protein-protein interaction network of the plant-virus pathosystem is very complicated. The presence of HC-Pro in chloroplasts indicated that there are other functions of HC-Pro besides its multiple functions in the cytoplasm. Previous work on virus-plant interactions mainly focused on the physiological changes in plants caused by viral infection^2^,^48^,^49^. PVY-infected plants show a reduced net photosynthetic rate^6^,^8^,^10^, but little is known about the molecular mechanisms involved in this reduction. Interactions between host plant proteins and viral components are presumed to play a vital role in these changes^47^.

The results presented here indicate that PVY HC-Pro can interact with the chloroplast CF_1_β-subunit in *Nicotiana tabacum*. The HC-Pro transgenic plants showed a reduced photosynthetic rate compared to wild-type plants, as was observed in PVY-infected plants. The interaction between HC-Pro and the NtCF_1_β-subunit led to a decreased number of chloroplast ATP synthase complexes in both the HC-Pro transgenic and PVY-infected tobacco. Although the HC-Pro/NtCF_1_β-subunit interaction did not affect the enzymatic activity of ATP synthase, the abundance of chloroplast ATP synthase was greatly reduced in both PVY-infected and HC-Pro transgenic plants. As a result, the total ATP synthase activity was decreased and the energy supply for photosynthesis may be strongly disturbed, leading to the reduced photosynthesis in PVY-infected plants. ATP synthase activity has been reported as a potentially limiting factor of photosynthesis^50^,^51^. Thus, the photosynthetic capacity of the PVY-infected plants was likely disrupted due to this interaction. The molecular approach applied in this study illuminated the phenomenon observed in PVY-infected plants and added a new function of HC-Pro besides its known multiple functions. The reduced photosynthetic rate is a comprehensive result of multiple host-virus interactions. The interaction between HC-Pro and the NtCF_1_β-subunit identified in this study is one of the causes of the decreased photosynthetic rate observed in potyvirus-infected plants. The mechanism underlying the impairment of photosynthesis in virus-infected plants has been explored for many decades. The disrupted activity of photosystem II (PSII) was previously thought to be the main cause of this reduced photosynthetic activity^4^,^7^,^52^,^53^. Our results presented here demonstrated some disturbance of photosynthetic rate correlated with ATP synthesis, thus deepening our understanding of viral attack on the photosynthesis system.

The chloroplast F-ATPase stromal region CF_1_ contains five subunits, α, β, γ, δ and ε, that are assembled in a 3:3:1:1:1 stoichiometry^54^. Here, we have shown that PVY HC-Pro can interact with the NtCF_1_β-subunit. However, HC-Pro does not bind directly to the ATP-binding site on the β-subunit. Rather, it binds to the N-terminus (residues 1–95) of the β-subunit. *In vitro* biochemical assays indicated that this interaction did not affect the enzymatic activity of chloroplast ATP synthase. The known inhibitors of ATP synthase are all small molecules or short peptides, such as singlet oxygen^55^, tentoxin^56^ and melittin^57^. The large HC-Pro protein would not fit into the inhibition sites for this enzyme, and this explains why HC-Pro did not affect the enzymatic activity of ATP synthase. On the other hand, the correct assembly of ATP synthase is the basis for its structure and function as a complete enzyme. HC-Pro interacts with the N-terminus of the β-subunit, which is important for its assembly into the CF_1_ complex^20^. Thus, the interaction between HC-Pro and the CF_1_β-subunit could disturb the assembly of ATP synthase and the level of ATP synthase would be reduced due to this interaction in PVY-infected tobacco.

Virus-plant interactions represent an interesting topic involving many different stages and changes that take place both in plants and viruses. The interactions between viral proteins and plant factors are key elements of viral infections, viral movements and the development of associated symptoms. The 11 PVY proteins have been shown to interact with different host proteins during different stages of viral infection^58^,^59^,^60^,^61^. We verified the interaction between HC-Pro and the NtCF_1_β-subunit here. And the biological significance of this interaction was further demonstrated. HC-Pro has no effect on the enzyme activity of assembled ATP synthase, instead, the amounts of ATP synthase in both the HC-Pro transgenic and PVY-infected tobaccco were significantly reduced. HC-Pro may interfere with the assembly of NtCF_1_β-subunit into the whole enzyme, leading to the reduced amount of ATP synthase and finally caused the reduction of photosynthetic rate. The findings presented here regarding the interaction of HC-Pro with the β-subunit of chloroplast ATP synthase have contributed to our understanding of one of the causes of the reduced photosynthetic rate observed in PVY-infected plants.

## Methods

### Plant material and growth conditions

Wild-type *Nicotiana tabacum* tobacco was inoculated with PVY virus according to Mojca Milavec^62^ with a few modifications. Gloved fingers were wetted with sap from the PVY-infected tobacco, and inoculation was carried out by gently rubbing the bottom leaves with tripolite. Sap from healthy tobacco was used as a mock inoculation control. The inoculated plants (PVY-inoculated) and corresponding control plants (wild-type and mock-inoculated) were grown in a constant incubator at 22 °C under a 16-h-light/8-h-dark photoperiod at the light intensity of 50 μmol m^−2^ s^−1^. PVY-infected plants at 12 days post-inoculation (dpi), together with control plants, were employed for future analysis.

Wild-type and HC-Pro transgenic *Nicotiana tabacum* plants were grown under standard greenhouse conditions at 25 °C under a 16-h-light/8-h-dark photoperiod at the light intensity of 100 μmol m^−2^ s^−1^. *N. tabacum* was transformed using *Agrobacterium tumefaciens* strain EHA105 through the leaf disc method. The transit peptide from the ribulose bisphosphate carboxylase small subunit (*rbcs*) was fused to the N-terminus of HC-Pro (the fusion was referred to as TP-HC-Pro) to enable its entry into the chloroplasts. The *TP-HC-Pro* gene was cloned into pCAMBIA-1302 vector through the restriction site *Nco*I/*Spe*I to generate the HC-Pro transgenic plants. The positive transformants were employed for future analysis.

### Subcellular localization of HC-Pro in Arabidopsis protoplasts

To test the efficiency of the transit peptide of *rbcs*, the *TP-HC-Pro* gene was cloned into the modified pE3025 vector (which contained GFP instead of RFP) using the *Sac*I/*Kpn*I site to form the pE3025-TP-HC-Pro vector to analyse the subcellular localization of HC-Pro. Rosette leaves of 4-week-old Arabidopsis plants grown under short-day conditions were used for isolation of protoplasts. The pE3025-TP-HC-Pro vector was transformed into Arabidopsis protoplasts according to a previously described protocol^63^. GFP fluorescence in the protoplasts was visualized using a confocal laser scanning microscope (LSM510, Carl Zeiss).

### Isolation of chloroplasts and extraction of chloroplast proteins

Intact chloroplasts were isolated using a chloroplast isolation kit (Sigma-Aldrich) according to the manufacturer’s instructions. Chloroplasts were collected after centrifugation at 4 °C and 1000 × g. Intact chloroplasts were separated from the broken chloroplasts by centrifugation on top of a 40/80% Percoll gradient. The band formed by intact chloroplasts at the interface between the 40% and 80% Percoll layers was collected and used for the following experiments. Chloroplast proteins were extracted with lysis buffer A (50 mM Tris-Cl, pH 8.0; 150 mM NaCl; 0.5% Triton; 1× Protease Inhibitor Cocktail; 1 mM PMSF) and diluted with the same volume of buffer B (50 mM Tris-Cl, pH 8.0; 150 mM NaCl; 1× Protease Inhibitor Cocktail; 1 mM PMSF). The lysate was centrifuged at 4 °C and 5000 × g for 10 min to separate the membranes and the supernatant. The chloroplast proteins in the supernatant were used for future analysis.

### Measurement of photosynthetic rates

The photosynthetic rates of wild-type, mock-inoculated and PVY-infected tobacco plants were measured using the LI-6400 XT Portable Photosynthesis System according to the user manual. Five values were collected at the light intensity of 600 μmol m^−2^ s^−1^ for each plant. Three biological repeats were done.

The photosynthetic rates of wild-type, empty-vector transgenic and HC-Pro transgenic plants were measured using the same method except that values were collected at different light intensity from 50 to 600 μmol m^−2^ s^−1^ to make the light intensity curve. Three independent lines were used for the measurement of photosynthetic rates, and three biological repeats were conducted for each line.

### Identification of the interaction between HC-Pro and NtCF_1_β-subunit in yeast

We used a yeast two-hybrid system to search for HC-Pro-interacting proteins in an *N. tabacum* cDNA library according to the user manual for the BD Matchmaker Library Construction and Screening Kit (BD Biosciences). The cDNA library was constructed by our lab previously using the mature leaves of *N. tabacum* L. cv. Xanthi NN about four weeks’ old^38^. The tobacco plants were grown under standard greenhouse conditions at 25 °C under a 16-h-light/8-h-dark photoperiod. HC-Pro was used as a bait to screen the library. In the obtained positive clones, the chloroplast CF_1_β-subunit (NtCF_1_β-subunit) was found to interact with HC-Pro. The full-length coding sequences of the *NtCF*_*1*_*β-subunit* was cloned into pGADT7 via the *EcoR*I/*Xho*I sites to form the pGADT7-NtCF_1_β-subunit plasmid. The reconstructed plasmid was co-transformed with the pGBKT7-HC-Pro vector described previously^36^, into *S. cerevisiae* AH109 cells to verify their interaction in yeast. Appropriate negative controls were produced at the same time.

### GST pull-down assay *in vitro*

The full-length coding sequence of *HC-Pro* was cloned into the pET-30a vector via the *EcoR*I/*Not*I sites to form the pET-30a-HC-Pro vector, and the full-length coding sequence of *NtCF*_*1*_*β-subunit* was cloned into the pGEX-4T-1 vector via the *EcoR*I/*Sal*I sites to form the pGEX-4T-1-NtCF_1_β-subunit plasmid. The proteins were induced at 16°C for 12–14 hours in *E. coli* BL21 cells. HC-Pro was expressed with a 6 × His tag (His-HC-Pro), and the NtCF_1_β-subunit was expressed with a GST tag (GST-NtCF_1_β). The purified GST-NtCF_1_β protein was bound to Glutathione Sepharose 4B (GE Healthcare) for 1 hour at 4 °C and then washed with lysis buffer (150 mM NaCl, 0.1 M Tris pH 8.0, 10% glycerol) three times. His-HC-Pro was added to the GST-NtCF_1_β-bound Sepharose and incubated at 4 °C for 2 additional hours. The Sepharose was then washed with the same lysis buffer three times. Samples were eluted from the Sepharose with the elution buffer (20 mM reduced glutathione in 50 mM Tris-Cl, pH 8.0) and prepared with 2× SDS loading buffer. The pulled down His-HC-Pro was detected with an anti-His antibody.

### Co-immunoprecipitation assay with the chloroplast proteins *in vivo*

The chloroplasts of wild type, empty-vector transgenic and HC-Pro transgenic plants were isolated using the same method mentioned above. The chloroplast proteins were also extracted as mentioned. The chloroplast proteins in the supernatant were incubated with anti-GFP antibody overnight at 4 °C. The immunocomplex was captured with washed Protein A Agarose (Millipore) at 4 °C for 2 hours. The agarose beads were collected and washed with ice-cold PBS buffer three times. Samples were prepared with 1× SDS loading buffer and detected using a CF_1_β-subunit antibody (Product AS05085, Agrisera).

### Identification of the necessary domains for HC-Pro/NtCF_1_β-subunit interaction

Three deletion mutants, HC-Pro1 (residues 98–456), HC-Pro2 (residues 1–298) and HC-Pro3 (residues 1–97), were previously designed in our laboratory to generate pGBKT7-HC-Pro1, pGBKT7-HC-Pro2 and pGBKT7-HC-Pro3, respectively^36^. Two deletion mutants, NtCF_1_β-subunit1 (residues 96–498) and NtCF_1_β-subunit2 (residues 1–380), were designed for the NtCF_1_β-subunit. The coding sequences of the two mutants were cloned into the pGADT7 vector via the *EcoR*I/*Xho*I sites to form the pGADT7-NtCF_1_β-subunit1 and pGADT7-NtCF_1_β-subunit2 constructs, respectively. To determine the necessary domains of the HC-Pro/NtCF_1_β-subunit interaction, the constructed deletion mutants were co-transformed with vectors harboring the full-length gene of the other protein into *S. cerevisiae* AH109 cells, and appropriate negative controls were produced at the same time.

### Detection of the enzymatic activity of ATP synthase

To determine the hydrolytic activity of ATP synthase, the F_1_ component of chloroplast ATP synthase was isolated from spinach according to the method described by Richter^64^, and ATP was added as the reaction substrate. Following incubation at 37 °C for 5 minutes, the reaction was terminated with trichloroacetic acid (TCA), and the amount of Pi produced was determined by spectrophotometry at a wavelength of 740 nm.

To perform photophosphorylation assays, thylakoid membranes were extracted from spinach. An assay mixture containing 100 mM tricine-NaOH (pH 8.0), 10 mM NaCl, 100 μM PMS, 10 mM MgCl_2_, 6 mM ADP and 2 mM KH_2_PO_4_ was added to the thylakoid membrane extract, and the reaction was initiated by illuminating the membrane with a halogen lamp in a tank of CuSO_4_ for 2 minutes. The reaction was terminated by turning off the light and then quickly adding 1 ml of 0.5 M TCA. The amount of Pi remaining in the reaction system was again determined by spectrophotometry at a wavelength of 740 nm.

### Immunogold labeling of HC-Pro, NtCF_1_-β subunit and ATP synthase

Samples were prepared from tobacco leaf tissue fixed in 2% paraformaldehyde and 1% glutaraldehyde. Following serial dehydration with ethanol, the samples were embedded in LR White resin as described previously^65^. Thin sections were cut using a Leica UC6i microtome. The grids were blocked with 1% BSA in PBS buffer for 2 hours. Then the grids were incubated with the primary antibody diluted in blocking buffer for 3 hours. After washing with PBS buffer three times for 10 minutes each, the grids were incubated with the gold-labeled secondary antibody diluted in PBS buffer for 1 hour. The same washing step was repeated. To enhance the contrast of the samples, the grids were stained with uranyl acetate for 15 minutes and then washed with milli-Q water three times for 10 minutes each.

The primary antibody used to count the number of ATP synthase was against the whole ATP synthase bought from Agrisera (Product AS08370). The secondary antibody was conjugated to 10-nm gold particles to label the ATP synthase. The anti-HC-Pro antibody was used to label HC-Pro in PVY-infected tobacco. The corresponding secondary antibody was conjugated to 10-nm gold particles. The 5-nm gold particle labeled secondary antibody was used to indicate the NtCF_1_-β subunit. The samples were viewed using a JEM-123O transmission electron microscope. For the counting of the number of ATP synthase, the chloroplast plan area and the number of gold particles were calculated using iTEM software, and the data were processed to indicate the particle number per area of the chloroplast.

### Quantitative western-blot analysis

Chloroplast protein concentration was determined by Bradford method. Twenty microgram protein were equal loaded. Protein samples were separated on a 12% SDS page and electroblotted to nitrocellulose membrane. The membrane was blocked for one hour with 5% nonfat milk in TBST and probed with anti-AtpA antibody against the essential α-subunit of CF_1_ to estimate the amount of ATP synthase according to Rott *et al.*^50^. The large subunit of Rubisco (RbcL) was used as a loading control to demonstrate equal input in all lanes. The levels of the AtpA and RbcL proteins were quantified using ImageJ software. The amounts of ATP synthase relative to RbcL in each lane were caculated and the differences among the samples were used to demonstrate a change of ATP synthase level.

## Additional Information

**How to cite this article**: Tu, Y. *et al.* Interaction between PVY HC-Pro and the NtCF_1_β-subunit reduces the amount of chloroplast ATP synthase in virus-infected tobacco. *Sci. Rep.* **5**, 15605; doi: 10.1038/srep15605 (2015).
